# The tumor innate immune microenvironment in prostate cancer: an overview of soluble factors and cellular effectors

**DOI:** 10.37349/etat.2022.00108

**Published:** 2022-10-31

**Authors:** Maria Teresa Palano, Matteo Gallazzi, Martina Cucchiara, Federico Dehò, Paolo Capogrosso, Antonino Bruno, Lorenzo Mortara

**Affiliations:** 1 Laboratory of Innate Immunity, Unit of Molecular Pathology, Biochemistry and Immunology, Istituto di Ricovero e Cura a Carattere Scientifico (IRCCS) MultiMedica, 20138 Milan, Italy; 2 Laboratory of Immunology and General Pathology, Department of Biotechnology and Life Sciences, University of Insubria, 21100 Varese, Italy; 3 Unit of Urology, ASST-Sette Laghi, Ospedale di Circolo e Fondazione Macchi, University of Insubria, 21100 Varese, Italy; Université Paris-Saclay, France

**Keywords:** prostate cancer, tumor immune microenvironment, innate immune cell polarization, cytokines

## Abstract

Prostate cancer (PCa) accounts as the most common non-cutaneous disease affecting males, and as the first cancer, for incidence, in male. With the introduction of the concept of immunoscore, PCa has been classified as a cold tumor, thus driving the attention in the development of strategies aimed at blocking the infiltration/activation of immunosuppressive cells, while favoring the infiltration/activation of anti-tumor immune cells. Even if immunotherapy has revolutionized the approaches to cancer therapy, there is still a window failure, due to the immune cell plasticity within PCa, that can acquire pro-tumor features, subsequent to the tumor microenvironment (TME) capability to polarize them. This review discussed selected relevant soluble factors [transforming growth factor-beta (TGFβ), interleukin-6 (IL-6), IL-10, IL-23] and cellular components of the innate immunity, as drivers of tumor progression, immunosuppression, and angiogenesis within the PCa-TME.

## Introduction

Prostate cancer (PCa), the most common non-cutaneous disease affecting the male population, still accounts as the first cancer for incidence in males. Metastasis still represents a major challenge for PCa patient’s survival: while patients with primary tumor are characterized by a 5-year survival of 99%, only the 22% of subjects with metastatic disease, whose bone accounts as the primary site for dissemination, experienced a 5-year survival [1, 2]. As multifocal pathology, PCa is characterized by large intratumor heterogeneity [3], a relevant hallmark that strongly impact both on the surrounding tumor microenvironment (TME), tumor immune microenvironment (TIME) and response to therapy [3, 4].

With the introduction of the concept of immunoscore [5, 6], PCa has been classified as a cold tumor, thus driving the attention in the development of strategies aimed at blocking the infiltration/activation of immunosuppressive cells [such as myeloid-derived suppressor cells (MDSCs), type-2 macrophage (M2)-like/tumor-associated macrophages (TAMs), T regulatory (Treg) cells], favoring the infiltration/activation of anti-tumor immune cells (such as natural killer (NK) cells, CD8^+^ T cells) [7, 8]. This concept clearly places the TIME as a crucial element of PCa, that still requires a deep characterization, to define therapies able in targeting the PCa-TIME.

Here, we reviewed and discussed selected major soluble factors [transforming growth factor-beta (TGFβ), interleukin-6 (IL-6), IL-10, IL-23] and cellular components of the innate immunity, as drivers of progression, immunosuppression, and angiogenesis within the PCa-TIME.

## Selected soluble factors in TIME in PCa

The TME is enriched of soluble factors [9], produced both by tumor cells, stromal [10] and immune-infiltrating cells [11–14] that strongly impact of the extremely heterogeneous cellular phenotypes and functions found in the TIME. These soluble factors are also relevant in regulating the aberrant/altered cell-to-cell and cell-to-extracellular matrix (ECM) interactions within the TME, regulating key process in tumorigenesis, such as tumor cell proliferation, angiogenesis, and immunosuppression, thus impacting on response to therapies.

### TGFβ

TGFβ a ubiquitously expressed cytokine, is directly involved in several pathophysiological processes both in development and adult life, ranging to tissue healing/repair, fibrosis, and cancers [15, 16]. TGFβ accounts as a master regulator in response to tissue injury inducing epithelial-to-mesenchymal transition (EMT), fibroblast activation, cell migration, and modulates immune response [17]. TGFβ promotes cell cycle arrest, apoptosis and differentiation, thus regulating the overall cell homeostasis [17]. TGFβ dysregulation has been found as a shared features in diverse cancers [18, 19], where it exerts different roles, as related to cancer stages [20–22]. At early stages, TGFβ suppresses tumor growth, acting as a tumor suppressor gene, while during latest stages and in metastasis TGFβ enhances tumor growth and promotes angiogenesis, migration, and invasion [23].

**Figure 1.**
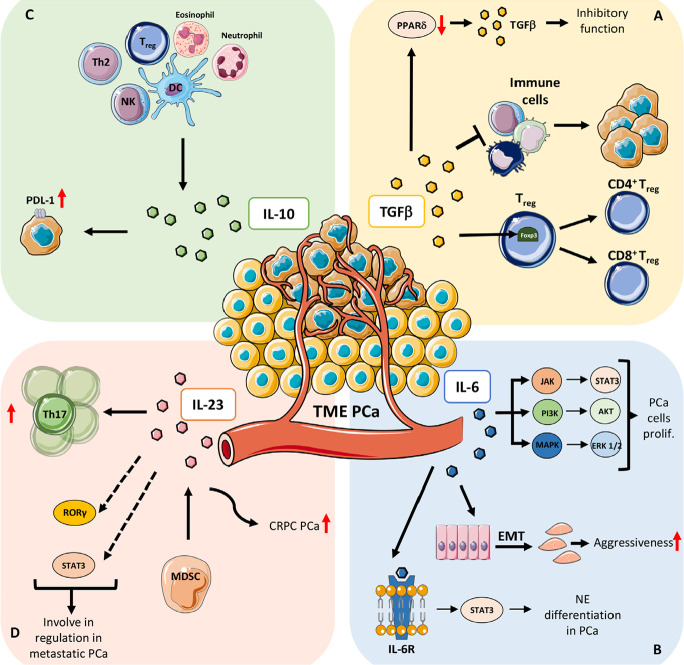
Mechanisms that involved soluble factors in PCa. A) TGFβ: *PPARδ*, a TGFβ target gene, play a crucial role in regulating TGFβ paradox in cancer (from oncosuppressor to a tumor-promoting factor); PPARδ repression increases the inhibitory effect of TGFβ on tumor cell while PPARδ induction promotes TGFβ pro-tumoral functions; one of the crucial roles of TGFβ in orchestrating TME is mainly focused on immune cells on which works as immune suppressor molecule thus sustaining immune pro-tumoral functions; TGFβ is involved in Treg cell development and differentiation by inducing Foxp3 expression, a specific marker of Treg subset that controls and maintains immune tolerance and homeostasis. B) IL-6: IL-6 mediated activation of signal transducer and activator of transcription (STAT)/Janus kinase (JAK) axis has been demonstrated to support PCa cell proliferation, via extracellular signal-regulated kinase 1 and 2 (ERK1/2)-mitogen activated protein kinase (MAPK) pathway, and the phosphoinositide 3-kinase (PI3-K) pathway; IL-6 play a major role in increasing PCa aggressiveness by instructing EMT and homing of metastatic clones to the bone; IL-6 receptor (IL-6R) signaling has been demonstrated to be crucial in favoring the neuroendocrine differentiation in PCa, by the canonical activation of STAT3 transcription factor. C) IL-10: IL-10 is expressed by several cell types of the immune system, including dendritic cells (DCs), NK cells [27–29], eosinophils, neutrophils, and T cell subsets. IL-10 also induces expression of neuroendocrine markers and programmed death-ligand 1 (PD-L1) in PCa cells. D) IL-23: IL-23 mediate expansion of Th17 cells and acts as a prognostic factor in patients with metastatic PCa; other mechanisms involving IL-23 as regulator of metastatic PCa, include the altered stimulation of the retinoic acid receptor-related orphan receptor gamma (RORγ) and STAT3 pathways; IL-23, produced by MDSCs, serve as promoter of castration-resistant prostate cancer (CRPC), by activating androgen receptor (AR) signaling and enhancing cell proliferation in a non-cell autonomous manner in PCa. prolif: proliferation; Th2: T helper 2; red arrows up and down: upregulation/increase and downregulation/decrease

The TGFβ target gene, peroxisome proliferator activated receptor delta (*PPARδ*) seems to play a crucial role in regulating TGFβ paradox in PCa, thus PPARδ repression increases the inhibitory effect of TGFβ on tumor cells, while PPARδ induction promotes TGFβ pro-tumoral functions (Figure 1A) [24]. In PCa cells, unresponsiveness to the TGFβ antiproliferative function [25] maybe due to the lack of TGFβ-receptor expression and correlates with high grade tumors [26].

Within TME, TGFβ is expressed and produced by different cell types, including tumor cells, tumor stroma, and infiltrating immune cells [23]. TGFβ acts as one of the most immunosuppressive factors in the TIME, further supporting tumor progression (Figure 1A). Immunosuppressive activities of TGFβ include inhibition of cell cytotoxicity induction of Treg cell development and differentiation, by inducing forkhead box p3 (Foxp3) expression, a specific marker of Treg subset that controls and maintains immune tolerance and homeostasis (Figure 1A) [27, 28]. Also, TGFβ induce the suppression of CD8^+^ T cell activity and support PCa growth and immunoescape [29]. TGFβ has been reported to support therapy hormonal resistance in PCa; of note, TGFβ blockade has been found to limit this effect, by inducing apoptosis in tumor cells, limiting angiogenesis and improving immune cell infiltration and anti-tumor immunity in PCa [30].

TGFβ has been reported to synergize with IL-6, IL-7, C-X-C motif chemokine ligand 8 (CXCL8)/IL-8 in promoting the EMT process, which is an essential phenomenon in metastasis formation [31–34]. TGFβ can support EMT and metastasis development, via AR. The silencing of AR in transgenic adenocarcinoma of the mouse prostate (TRAMP) animals, has been reported to support EMT, by reducing Epithelial-cadherin (E-cadherin) expression and increasing vimentin and Neural-cadherin (N-cadherin) expression [35]. AR knock-down increases cell migration and metastasis formation, in a TGFβ dependent manner [35]. On the other way, TGFβ suppression could lead to the up-regulation of ERK which could stimulate EMT-dependent migration and invasion of PCa cells [36].

Increased level of circulating TGFβ is associated with a worse prognosis in PCa patients [37]. Within prostate tissue, high expression of TGFβ is linked to poor prognosis while lower expression is associated with benign tumors [38].

The role of TGFβ in PCa growth and progression exerts a complex and wide action on both tumor cells and microenvironment suggesting the use of this molecule as positive (with enhancing therapy) and negative (inhibition) regulator of TME depending on/according to the tumor stages and landscape.

### IL-6

IL-6 is a pleiotropic pro-inflammatory cytokine largely expressed in PCa. IL-6 can be expressed both by the tumor, stromal and immune compartments in PCa [39–41]. Major effects of IL-6 include its abilities to regulate cell proliferation, cell differentiation, apoptosis, inflammation, and angiogenesis [39–41]. As established major soluble mediator of inflammation, IL-6 is crucial in governing cancer-related inflammation, including in PCa [41, 42].

IL-6 account as a major activator of the signaling pathway of JAK and STAT3, thus acting as a master regulator within the PCa TME [39, 43, 44]. IL-6 mediated activation of JAK/STAT axis has been demonstrated to support PCa cell proliferation, via ERK1/2-MAPK pathway, and the PI3-K pathway (Figure 1B) [45]. IL-6 has been found to synergize with oncostatin-M (OSM) in promoting PCa aggressiveness and malignancy via PI3K/AKT pathway *in vivo* and in PCa human tissues [45].

Also, IL-6 play a major role in increasing PCa aggressiveness by instructing EMT and homing of metastatic clones to the bone (Figure 1B). Also, aggressiveness and recurrence of PCa has reported to correlate with IL-6 polymorphisms [46]. Elevated serum levels of IL-6 have been detected in patients with untreated metastatic or CRPC, thus negatively correlating with tumor survival and response to chemotherapy. IL-6 is also implicated in the transition from hormone-dependent to CRPC, by transactivation of the AR.

In a study performed on 74 PCa patients, Nakashima et al. [47] found that serum IL-6 significantly correlated with the clinical stage of PCa, as recently confirmed by Zhou et al. [48] in a study showing that plasma IL-6 and TNFα levels significantly correlate with grading changes in localized PCa. IL-6R signaling has been demonstrated to be crucial in favoring the neuroendocrine differentiation in PCa, by the canonical activation of STAT3 transcription factor (Figure 1B) [49].

### IL-10

IL-10 is a cytokine characterized by its pleiotropic effects in immunoregulation and inflammation [50–52]. IL-10 has a central role during infection, by limiting the immune response to pathogens and thereby preventing damage to the host [53]. IL-10 was initially described as Th2-type cytokine [54]; further studies clearly demonstrated production of IL-10 was associated with tolerant or Treg cell responses. It is now well consolidated that IL-10 is expressed by many cells of the immune system, including DCs [55–57], NK cells [58–60], eosinophils [61, 62], neutrophils [63, 64], and all the T cell subsets (Th1, Th2, Th17, Treg, CD8^+^ T cells) (Figure 1C) [65–69]. By its anti-inflammatory and immunosuppressive activities, IL-10 support tumor progression, limiting efficient anti-tumor response [70–72].

IL-10 has been detected as elevated serum samples of PCa patients and has been correlated with poor prognosis and positively correlated with Gleason score [73]. Also, IL-10 and heat shock protein 90 (HSP90) expression revealed a highly significant correlation in advanced Gleason grading and tumor, node, and metastasis (TNM) staging cases of PCa [73]. A meta-analysis performed by Shao et al. [74] investigated the relation with IL-10 polymorphism and PCa, based on the fact that three common polymorphisms in the promoter of *IL-10* gene, −1082 A > G, −819 C > T, and −592 C > A, have been implicated to alter the risk of PCa [74] that have been considered as a controversial issue. The authors concluded that *IL-10* −1082 A > G, −819 C > T, and −592 C > A polymorphisms show significant evidence to be associated with PCa risk [74]. Therefore, L-patients carrying the *IL-10* −819 C > T and −592 C > A might develop a highly aggressive PCa [74].

Finally, Samiea et al. [75] recently demonstrated that IL-10 induces expression of neuroendocrine markers and PD-L1 in PCa cells, by supporting tumor cell survival by interaction with PD-1, and favoring immunosuppression (Figure 1C).

### IL-23

IL-23 is a heterodimeric cytokine consisting of two subunits, IL-12B and IL-23A, that belongs to the IL-12 group of cytokines. It is now largely demonstrated that the balance between the proinflammatory cytokine IL-12 and IL-23 in tumors is crucial in shaping the development of anti-tumor or pro-tumor immunity [76]. IL-23 was found to be overexpressed in many human tumors, including lung [77–79], colorectal [80–82], breast [83], ovarian [84], pancreatic [85], prostate [86], bladder [87] cancers, and multiple myeloma [88].

IL-23 has been reported to repress the level of cell senescence, induced by the AR antagonist enzalutamide and darolutamide, in CRPC cells [89]. Calcinotto et al. [86] found that MDSCs and IL-23 concentration increase in peripheral blood and tumor tissues from patients with CRPC. The authors also demonstrated that IL-23, produced by MDSCs, serves as promoter of CRPC, by activating AR signaling and enhancing cell proliferation in a non-cell autonomous manner in PCa (Figure 1D) [86]. Treatments able in blocking IL-23 were effective in contrasting MDSC-mediated resistance to castration and synergize with standard therapies in PCa [86]. Other mechanisms involving IL-23 as regulator of metastatic PCa, include the altered stimulation of the RORγ and STAT3 pathways (Figure 1D). Liu et al. [90] reported that IL-23 mediate expansion of Th17 cells and acts as a prognostic factor in patients with metastatic PCa (Figure 1D). Also, IL-23^+^ cells have been found to increase in PCa tissues and correlates with disease progression, as confirmed by The Cancer Genome Atlas (TCGA)-prostate adenocarcinoma (PRAD) cohort analysis [90]. TCGA-PRAD analysis also revealed that IL-23 expression associates with poor survival and CRPC-free survival. Increased presence of IL-23^+^ cells has been reported in PCa metastatic lesions as compared to non-metastasized ones [90]. Concerning the PCa therapeutic treatments, authors found that IL-23^+^ cells can predict poor clinical outcomes in patients receiving the abiraterone treatment, while no similar effect was observed in patients undergoing docetaxel treatment [90].

## Tumor innate immune microenvironment in PCa

The TME is characterized by extreme heterogeneity in cellular composition, that includes tumor cells and diverse cells of the host, such as cancer associated fibroblasts (CAFs), normal fibroblasts (NFs), endothelial cells (ECs) of the new generated blood vessels, and cells of both innate and adaptive immune system [91]. Here we focused our attention on the activities of selected innate immune cells found in the PCa tumor innate immune microenvironment (TIIME).

### Mast cells

Mast cells (MCs) are innate immunity effector cells primarily involved in the inflammatory response and allergy [92, 93]. The identification of tumor-infiltrating MCs dates to late 19th century [92, 93]. Studies examining both human cancer tissues as well as using experimental models show that MCs can exert either anti-tumor or pro-tumor activities. This dual role is strictly regulated by the tumor type, MC interactions with microenvironmental signals and with neighboring cells [85]. Apart for their “canonical role”, MCs have been reported to be able to produce several factors that can support tumor growth, such as CXCL8/IL-8, vascular endothelial growth factor (VEGF), platelet-derived growth factor (PDGF), nerve growth factor (NGF), stem-cell factor (SCF), together with matrix metalloproteases (MMPs), necessary for the ECM remodeling, thus favoring metastasis [94–96].

**Figure 2.**
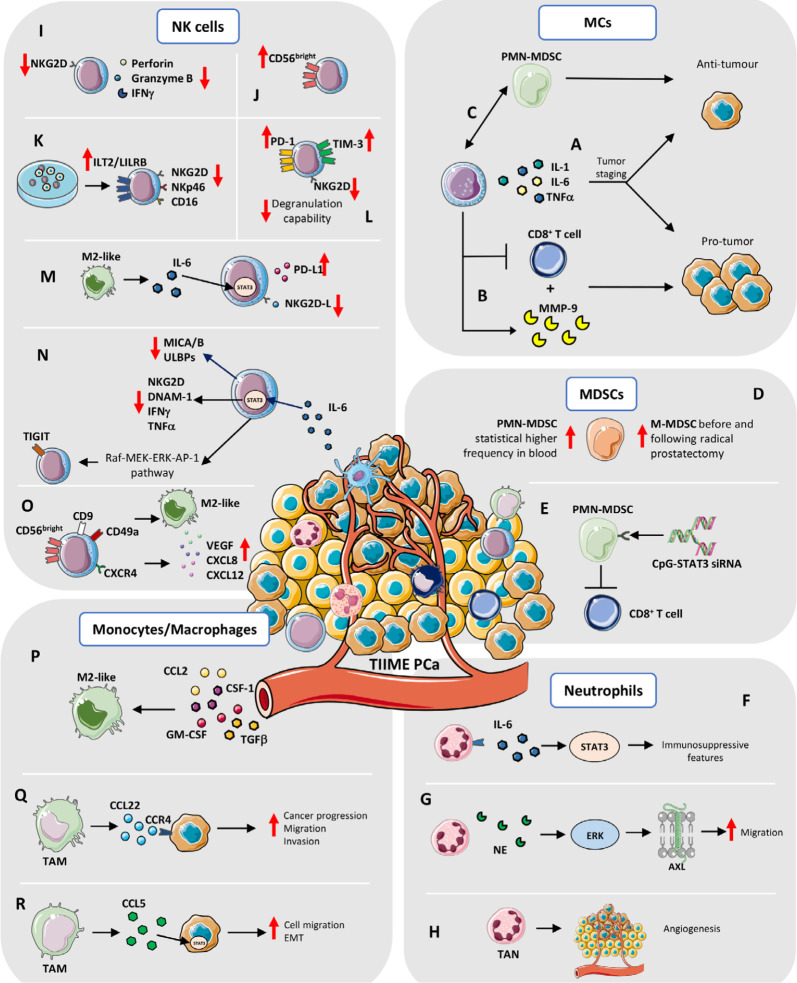
TIIME in PCa. MCs: A) The Janus behavior by MCs (production of IL-1, IL-6, and TNFα) depends on tumor staging: in early phase tumors, MCs acquire pro-tumorigenic properties; they became protective in late-stage cancer. B) MCs exert pro-tumor activities by producing MMP-9 and suppressing CD8^+^ T cell response. C) MCs have been found to acquire protective functions, via crosstalk with PMN-MDSCs, by interfering with *de novo* generation of neuroendocrine tumors. MDSCs: D) Brusa et al. [99] showed that circulating monocytes-MDSCs (M-MDSCs) increase in frequency, before and following radical prostatectomy, whereas Hossain et al. [100] showed higher frequency of blood PMN-MDSCs in PCa patients. E) Targeting Toll-like receptor 9 (TLR9)^+^ PMN-MDSCs, by STAT3 silencing, with cytosine-phosphate-guanine (CpG)-STAT3 small interfering RNA (siRNA) conjugate, Hossain et al. [100] found that this approach is successful on blocking the immunosuppressive activity *in vitro* of MDSCs on CD8^+^ T cells of PCa patients. Neutrophils: F) Neutrophils within the PCa TME, expressed IL-6R and the high amount of IL-6 in TME induces STAT3 mediated activation of immunosuppressive features. G) An *in vitro* study showed that neutrophil elastase (NE), a serine protease stored in neutrophils, induces ERK signaling in a dose dependent manner and activation of the AXL receptor tyrosine kinase (AXL) in PCa cell lines which showed increased migratory capability. H) Tumor-associated neutrophils (TANs) can also influence angiogenesis within TME of PCa and metastasis. NK cells: I) PCa tumor infiltrating NK are characterized by reduced expression of the activation receptor NK Group 2D (NKG2D), together with impaired degranulation capabilities and reduced production and release of cytolytic molecules, such as perforin, granzymes, and interferon gamma (IFNγ). J) Tumor infiltrating NK cells in PCa patients are enriched in immature CD56^bright^ cells. K) PCa cancer cells support the expression of Ig-like transcript 2 (ILT2)/leukocyte immunoglobulin like receptor B (LILRB) inhibitory receptors, together with downregulation of NKG2D and NKp46 and CD16 on NK cells. L) PCa circulating NK cells were found to increase their expression of PD-1 and T-cell immunoglobulin mucin family member 3 (TIM-3; as cell exhaustion markers), together with decreased level of NKG2D and degranulation capabilities, compared to circulating NK cells from control subjects. M) M2-like/TAMs in tumor environment limit NK cells cytotoxicity against metastatic CRPC (mCRPC) cells, by enhancing the PD-L1 levels and reducing NKG2D ligands production, through the IL-6-STAT3 pathway. N) IL-6, another abundant cytokine present both at tissue and systemic levels in PCa patients, limit NK cell anti-tumor activities, via STAT3 activation, by decreasing major histocompatibility complex-class I chain related proteins A and B (MICA/B) and UL16 binding proteins (ULBPs) NK cell-activating ligands, resulting in decrease NK cell killing capabilities. STAT3 activation in NK cells also results in reduced expression of activating receptors NKG2D, DNAX accessory molecule-1 (DNAM-1), IFN*γ*, and TNF*α* secretion. STAT3 was found to activate the rapidly accelerated fibrosarcoma (Raf)-mitogen-activated ERK kinase (MEK)-ERK-activator protein-1 (AP-1) pathway, which directly induces the expression of the T cell immunoreceptor with Ig and ITIM domains (TIGIT), in NK cells. O) NK cells isolated from peripheral blood of PCa patients acquire the CD56^bright^CD9^+^CD49a^+^(C-X-C motif chemokine receptor 4) CXCR4^+^ decidual-like phenotype and exhibit pro-angiogenic functions, inducing tube formation by endothelial cells, due to increased production of VEGF, CXCL8, CXCL12 and M2-like/TAM polarization. Monocytes/Macrophages: P) The M2-like TAM phenotype is driven by different stimuli within TME which include C-C motif chemokine ligand 2 (CCL2), colony stimulating factor 1 (CSF-1) as well as granulocyte-macrophage CSF (GM-CSF) and TGFβ, produced by cancer and stromal cells, that strongly contribute to macrophages polarization and in the generation of an immunosuppressive environment, via CXCL12 and IL-6. Q) Cancer cell/macrophage crosstalk is also driven to the opposite direction, as TAMs promote cancer progression, by stimulating migration and invasion, trough CCL22-C-C motif chemokine receptor 4 (CCR4) axis activation. R) TAM-derived CCL5 activates STAT3 signaling in cancer cells and increases cell migration, EMT, and cell invasion, as well as supports cancer stem cell self-renewal. Red arrows up and down: upregulation/increase and downregulation/decrease

MC-mediated anti-tumor activities relate to their ability to produce IL-1, IL-6, TNFα that induce apoptosis in tumor cells, together with chondroitin sulfate, that could exert a decoy activity by inhibiting metastases [97]. This dual behavior by MCs has also been observed in PCa, depending on tumor staging. While in early phase tumors MCs acquire pro-tumorigenic properties, they became protective in late-stage cancer, particularly in the case of the highly aggressive neuroendocrine PCa (Figure 2A). In PCa, MCs have been found to be enriched in areas of well-differentiated (WD) adenocarcinoma but not around poorly differentiated foci coexisting in the same tumors [98]. Of notice, while MCs exert pro-tumor activities in WD adenocarcinomas, by producing MMP-9 [96] and suppressing CD8^+^ T cell response [96] (Figure 2B), via crosstalk with polymorphonuclear (PMN)-MDSCs, MCs have been found to acquire protective functions by interfering with *de novo* generation of neuroendocrine tumors [94, 96, 97] (Figure 2C).

In a study performed using the H-subline of the Dunning tumor (Dunning-H) and angiotensin II type-1 (AT-1) models of PCa, Johansson et al. [101] found that intra-tumoral and peri-tumoral MCs have completely different behavior. In this study, while intertumoral MCs negatively regulate angiogenesis and tumor growth, peritumoral MCs were found to support PCa expansion. Moving to the human setting, the authors observed that patients with increased frequency of MCs in in the non-malignant stroma associated with poor prognosis in a significantly statistic manner [101]. Finally, the authors found that castration therapy increase MCs recruitment [101].

### MDSCs

MDSCs represent a heterogeneous immature myeloid cell population endowed with immunoregulatory functions and in particular inhibitory features against CD8^+^ cytotoxic T cells and NK cells in the TME of different types of cancers [102, 103]. Moreover, MDSCs are also involved in tumor angiogenesis and metastasis [104]. MDSCs were originally identified, in mice, as immature myeloid cells co-expressing granulocyte antigen type 1 (Gr-1) and CD11b surface markers [102]. Subsequently, murine MDSCs were characterized as two distinct subpopulations based on differences in their morphology and surface marker expression: cells resembling to granulocytic PMN cells, termed PMN-MDSCs, and cells with features shared with monocytes, named M-MDSCs. In mice, PMN-MDSCs are defined as CD11b^+^ lymphocyte antigen 6 complex locus C (Ly6C)^low^ lymphocyte antigen 6 complex locus G (Ly6G)^+^ cells, whereas M-MDSCs as CD11b^+^Ly6C^high^Ly6G^–^. In humans PMN-MDSCs are identified as CD11b^+^CD14−CD15^+^ cells or CD11b^+^CD14−CD66b^+^ cells, and M-MDSCs as CD11b^+^CD14^+^ major histocompatibility complex, class II, DR (HLA-DR)^−/low^CD15^−^ cells [104]. Mechanisms involved in MDSCs-dependent immune regulation are multiple and include depletion of arginine by arginase-1 (ARG1), release of nitric oxide (NO) by the inducible NO synthase (iNOS), and production of reactive oxygen species (ROS). Moreover, these cells exert indoleamine 2,3-dioxygenase (IDO) enzyme activity causing tryptophan elimination and induction of kynurenine inhibitory metabolite and activation of Treg cells by IL-10 and TGFβ production [105–107].

Patients with PCa have increased circulating and tumor infiltrating MDSCs. Brusa et al. [99] showed that circulating M-MDSCs were augmented before and following radical prostatectomy, whereas Hossain et al. [100] reported increased frequency of circulating PMN-MDSCs in PCa patients (Figure 2D), compared to healthy subjects, and this increase turned out to be more than double in the mCRPC patients. Moreover, Idorn et al. [108] found that circulating M-MDSCs increase in patients with CRPC, together with increased number of Treg cells, correlating with negative prognosis and with a shorter median overall survival (OS). High numbers of intratumor MDSCs have been also reported in patients, that do not respond to androgen deprivation therapy [86].

Given the close dependence of these cells on STAT3 signaling, Hossain et al. [100] generated a CpG-STAT3 siRNA conjugate that, by targeting TLR9^+^ PMN-MDSCs limits the immunosuppressive activity of MDSCs on CD8^+^ T cells of PCa patients, *in vitro* (Figure 2E). At the same time, in different mouse models of PCa it has been demonstrated the role of CD11b^+^Gr-1^+^ MDSCs in tumor initiation and progression [99]. As also showed by Calcinotto et al. [86] in several murine models of PCa, including the phosphatase and tensin homolog (*PTEN*) conditional knockout (KO) and TRAMP-C1 mouse models, PMN-MDSCs can activate the AR pathway by IL-23 release and favor tumor cell proliferation even after androgen inhibition. Antibody-mediated blockade of IL-23 or IL-23 receptor can counteract MDSCs’ effects on resistance to castration and restore androgen deprivation therapy. The interplay between IL-23 and MDSCs is in line with observation in humans, since CRPC patients showed both elevated levels of IL-23 and increased number of MDSCs in blood and tumor [86]. It has been also shown that PCa-derived CXCL5 can recruit CXCR2-expressing MDSCs in a mouse model of PCa and inhibition of MDSCs through blocking of CXCL5-CXCR2 axis can restore anti-tumor activities [109].

Therefore, given the implications and involvement of MDCSs in PCa pathology, these cells have become central to the study of new therapeutic approaches for PCa and/or CRPC [110].

### Neutrophils

Neutrophils are professional phagocytes of the innate immunity, are primarily involved in early host defense form pathogens and induction of acute inflammation [111, 112]. Neutrophils can release lytic enzyme, produce ROS and generate neutrophil extracellular traps (NETs) [113]. Neutrophils are found as tumor-infiltrating cells (known as TANs) within the TIME [113], where they can be polarized towards the anti-tumor type-1 neutrophil (N1) subset, that promotes T cell-mediated tumor clearance, or pro-tumor N2-subsets, which act as immunosuppressive cells [113]. This phenotypic and functional switch of TANs could be linked to tumor stages and TME. Indeed, TANs switch and polarization is regulated by TGFβ, that induces tumor promoting N2 phenotype while, blockage of TGFβ stimulates anti-tumor function of TANs [113].

In PCa TIME, neutrophils expressed IL-6R (CD126) and the high amount of IL-6 induces STAT3 signaling that regulates immunosuppressive features (Figure 2F) [114]. An *in vitro* study showed that NE, a serine protease stored in neutrophils, activates ERK signaling in a dose dependent manner and the AXL receptor tyrosine kinase in PCa cell lines, that acquired increased migratory capability (Figure 2G). TANs can also influence angiogenesis within PCa-TME and can support metastasis (Figure 2H). Using prostate cancer cells type 3 (PC-3) cell line, orthotopically injected in non-obese diabetes (NOD)/severe combined immunodeficiency (SCID) mice, it has been showed that both neutrophils and TANs are able to secrete higher amount of MMP-9, compared to macrophages and TAMs [115] and TANs-derived MMP-9 supports metastasis development [115]. Neutrophil function is modulated by microenvironmental and cancer-derived stimuli, such as sialic acid binding immunoglobulin like lectin (Siglec) ligands, which are upregulated in many cancers, including PCa [116]. These ligands can bind to the inhibitory CD33-related Siglecs and exert a negative immunomodulatory function. The lectin galactoside-binding soluble 3 binding protein (LGALS3BP), a ligand for human Siglec9, is upregulated in the ECM of PCa specimens and can inhibit neutrophils activation, supporting immune escape of cancer cells [116]. Diverse studies underlined a correlation between circulating neutrophils [in terms of neutrophils-to-lymphocytes ratio (NLR)] in PCa patients, and patients features as elevated NLR is associated with shorter OS in mCRPC subjects [117], while lower NLR in post-chemotherapy mCRPC patients is associated to longer OS [118]. The NLR value resulted to be increased also comparing PCa and benign prostatic hyperplasia (BPH) patients and is predictive of biochemical recurrence in patients with localized PCa after radical prostatectomy [117–120]. These findings suggest a role of circulating neutrophils and TANs in determining disease progression and cancer development that still need to be fully elucidated.

### DCs

DCs are known as the most powerful antigen presenting cells (APCs), being able to activate T cells but also to drive innate immune cells. DCs consist of three major cell subpopulations: myeloid conventional DCs1 (cDCs1), myeloid cDCs2, and plasmacytoid DCs (pDCs) [121]. cDCs1 exert the most potent anti-tumor functions resulting from the ability to release IL-12 and orchestrate anti-tumor CD8^+^ T cell effectors functions, through cross-presentation and induction of anti-tumor CD4^+^ Th1 type cells [121].

Several experimental evidence has suggested the anti-tumor role played by DCs in PCa, however, during cancer development these cells appeared reduced in number and dysfunctional or immature, favoring a tolerogenic environment [122–124]. Moreover, in PCa TME, it has been reported that VEGF was able to inhibit antigen presentation by DCs [125]. Given the potential therapeutic use of DCs, the first DC therapy was approved by the Food and Drug Administration (FDA) in 2010, the Sipuleucel-T in patients with the mCRPC [126]. A portion of mCRPC patients treated with this DC-based immunotherapy experienced improved OS, however most potent vaccines or combination therapies are needed to counteract the PCa immunosuppressive microenvironment and to implement immunotherapy [127].

### NK cells

NK cells are large granular lymphocytes characterized by natural cytotoxicity against cancer cells, together with cytokines-producing effector functions [128–131]. NK cells represent the 10–15% total human peripheral blood mononuclear cells [128–131]. NK cells discriminate between healthy self-cells and infected or tumor cells trough activating/inhibiting receptors present on cellular membrane and their major histocompatibility complex (MHC) class I–specific receptors that finely regulate NK cells killing activity [128–130]. NK cells recognize both self-ligands on stressed cells such as ULBP and MIC molecules and non-self-ligands, as well as TLR ligands, that instruct the production of IFNγ and cytotoxicity by NK cells [128–130]. Moreover, NK cells can eliminate antibody-coated cells through the antibody-dependent cell cytotoxicity (ADCC) enabled by the expression of fragment crystallizable (Fc) receptor CD16 on the cell surface [128–130].

Depending on expression of the neural cell adhesion molecule (NCAM), namely CD56, and the low-affinity Fc receptor CD16, human NK cells exhibit different phenotype and functionalities and can be classified into two major cell subsets [128–130]. CD56^dim^CD16^+^ NK cells constitute the 85–90% of both peripheral blood cytolytic NK cells, while cytokines-producing CD56^bright^CD16^–^ account as the 10–15% of circulating NK cells [128–130]. While CD56^dim^CD16^+^ NK cells express CXCR1 to allow their recruitment to peripheral inflammation area [132], CCR7 was found expressed on CD56^bright^CD16^–^ to permit NK cells homing towards lymph nodes [133].

Within the developing decidua, a third NK cell subset has been found, defined as CD56^brigtht^CD16^–^ NK cells, characterized by tolerogenic functions for the developing fetus, together with pro-angiogenic functions, these latter necessary for the correct development of spiral artery [134, 135].

NK cells have been found altered in their phenotype and functions in diverse solid and hematological cancers [136, 137]. In solid cancers, hypofunctional NK cells have been found both at tumor tissue and peripheral levels [136–139]. As shared features of cell anergy in cancers, NK cells have been reported have decreased levels of NKG2D (a major activator receptor), together with impaired degranulation capabilities and reduced production and release of cytolytic molecules, such as perforin, granzymes, and IFNγ (Figure 2I) [136, 140]. NKG2D-deficient TRAMP mice exhibit three times fold increase in developing aggressive poorly differentiated prostate carcinoma, compared to NKG2D wild type (*wt*) TRAMP animals [141]. Moreover, in NKG2D *wt* TRAMP mice, progression to PD PCa was mostly associated with downregulation of NKG2D ligand expression by tumor cells [141].

Several soluble factors present in the TME [142, 143], such as TGFβ, IL-6, adenosine (after hypoxia), prostaglandin E^2^ (PGE^2^), act as relevant players in shaping NK cell activities, including PCa. Also, the strong immunosuppressive microenvironment characterizing PCa impairs NK cell functions at multiple levels [144].

In a first study, Pasero et al. [145] traced NK cells activities in the peripheral blood of patients with metastatic PCa, with 5 year-follow-up. Authors observed that PCa patients with longer time of castration response and OS displayed increased expression of activating receptors and high cytotoxicity by NK cells [145]. Natural cytotoxicity receptors (NCRs) NKp30 and NKp46 were found as the most predictive markers of OS and time to castration resistance in the cohort of patients analyzed [145]. Together, these results place NK cells as potential predictive biomarkers for the stratification of PCa patients having longer time of castration response, thus paving the way to explore therapies aimed at enhancing NK cells in metastatic PCa patients. Another study by Pasero et al. [144] showed that tumor infiltrating NK cells in PCa patients are enriched in immature CD56^bright^ cells (Figure 2J) that, while expressing markers of activation, are poorly cytotoxic and that TGFβ, an immunosuppressive cytokine abundant in PCa tissues, strongly regulate this process. By performing NK cell-PCa cells co-culturing experiments, the authors showed that PCa cancer cells support the expression of ILT2/LILRB inhibitory receptor, together with downregulation of NKG2D, NKp46, and CD16 on NK cells, negatively impacting on NK-tumor cell recognition (Figure 2K) [144]. Interestingly, NKp46 was also reduced in PCa circulating NK cells [144].

A study by Koo et al. [146] reported that reduction of CD56^bright^CD16^−^ NK cells precede NK cell dysfunction in PCa patients. Authors observed that NK cell activation and the proportion of CD56^bright^ NK cells were lower in PCa patients, compared to control subjects. Also, increased CD56^dim^ to CD56^bright^ ratio was detected in PCa patients that gradually increased in association with tumor staging [146].

The JAK/STAT signaling is involved in PCa tumor suppression [147]. Combined inhibition of JAK1,2/STAT3-PD-L1 signaling pathways has been found to suppress CRPC immune escape to NK cell anti-tumor activities [147].

In a study on 43 subjects undergoing prostate biopsy and using a liquid biopsy-based method, Barkin et al. [148] observed that low subjects with levels of NK cell activity were more likely to have a positive outcome at prostate biopsy.

PCa circulating NK cells were also found to increase their expression of PD-1 and TIM-3 (as cell exhaustion markers), together with decreased level of NKG2D and degranulation capabilities, compared to circulating NK cells from control subjects (Figure 2L) [149]. Also, PCa circulating NK cells were found to increase their production of monocyte recruiting and macrophage polarizing factors that resulted in their capabilities to increase monocyte migration and M2-like/TAMs polarization, compared to circulating NK cells from healthy donors [149].

The relevance of monocyte/macrophage-NK cell interactions in PCa has been demonstrated in a study showing that M2-like/TAM phenotype in tumor environment limit NK cells cytotoxicity against mCRPC cells, by enhancing the PD-L1 levels and reducing NKG2D ligands production through the IL-6/STAT3 pathway (Figure 2M) [150].

IL-6, another abundant cytokine present both at tissue and systemic levels in PCa patients, limits NK cell anti-tumor activities, via STAT3 activation, by decreasing MICA/B and ULBPs NK cell-activating ligands, resulting in decrease NK cell killing capabilities. STAT3 activation in NK cells also results in reduced expression of activating receptors NKG2D, DNAM-1, IFN*γ*, and TNF*α* secretion [151]. However, IL-6 was demonstrated to not favor the decidual like CD56^bright^CD9^+^CD49a^+^NKG2D^low^ phenotypic switch in healthy donor-derived NK cells. Finally, STAT3 was found to activate the Raf-MEK-ERK-AP-1 pathway which directly induces the expression of the TIGIT (Figure 2N) receptor belonging to the poliovirus receptor (PVR) family CD155, found increased in CRPC patients, resulting in poor survival [152] and high-risk recurrence after radical surgery [152].

Pro-angiogenic decidual-like NK (dNK-like) cells, characterized by the CD56^bright^CD16^–^VEGF^high^CXCL8^+^IFN^low^ subset, has been found in tumor infiltrating and circulating NK cells in NSCLC [153], pleural effusion of patients with metastatic cancers [154] and CRC patients [155]. These dNK-like cells have been found to be induced by TGFβ, as also confirmed by experimental *in vitro* models of TGFβ polarized cytolytic NK cells [149, 153, 154, 156, 157].

Recently, Gallazzi et al. [149] demonstrated that NK cells isolated from peripheral blood of PCa patients are polarized towards the CD56^bright^CD9^+^CD49a^+^CXCR4^+^ decidual-like phenotype and exhibit pro-angiogenic functions, inducing tube formation by endothelial cells, due to increased production of VEGF, CXCL8, CXCL12 by NK cells and their ability to polarize macrophages toward the M2-like/TAM phenotype (Figure 2O) [149].

### NK T cells

NK T (NKT) cells represent heterogeneous innate-like T lymphocytes in both human and mice, that co-express both T cell receptor (TCR) and NK cell markers. NKT cells are able to recognize lipid antigens through CD1d molecule. NKT cells include two different subpopulations: type I and type II NKT cells, according to TCR rearrangements and glycolipid reactivity [158, 159]. Type I or invariant NKT (iNKT) cells can be stimulated by alpha-galactosylceramide (α-GalCer) and have an invariant TCRα chain rearrangement, while TCRβ chains present a restricted repertoire, and consist of three cellular subsets, named NKT1, NKT2, and NKT17, with similarities to Th1, Th2, and Th17 cell subsets, respectively. Type II NKT cells, are characterized by a higher variable repertoire of variable alpha region (Vα) rearrangements [160].

iNKT cells were found to be key active anti-tumor effectors [161], whereas type II NKT cells, have rather a pro-tumor role, promoting growth and metastasis [160]. iNKT cells exert anti-tumor effector cell activities by producing several Th1 cytokines, i.e. IFNγ, TNFα, and by eliminating CD1d-expressing tumor cells, thus they represent a potential intriguing therapeutic cellular tool against cancer development and metastasis [162]. In addition, in patients with advanced PCa with elevated prostate-specific antigen (PSA) levels, peripheral blood iNKT cells were decreased in comparison to PCa patients with androgen withdrawal and stable PSA levels [163]. Also, in patients with androgen-independent advanced PCa, peripheral blood iNKT cell frequency was reduced [164], and results from *in vitro* activated iNKT with α-GalCer and autologous-irradiated PBMCs for 3–4 weeks, showed that PCa patients had iNKT with defective IFNγ production, compared to healthy controls, whereas IL-4 secretion was normal [164].

In the spontaneous TRAMP model, iNKT cells infiltrate prostate tumor via CCL2/CCR5 pathway; however, tumor cells only partially activate iNKT cells, because of their impairment to release IFNγ [165]. Of note, this defect could be reverted both *in vitro* and *in vivo* by using combining IL-12 and α-GalCer [165]. Interestingly, studying Jalpha18 (Jα18)^–/–^ mice, selectively deficient in iNKT cells, Bellone et al. [166] generated male TRAMP Jα18^–/–^ mice, and found that tumor onset was accelerated and more aggressive comparing to TRAMP mice, indicating that iNKT play a relevant role in the immune surveillance of spontaneous TRAMP model.

Finally, in the TRAMP model, iNKT were able to interact with TAMs in the TIME, kill pro-angiogenic tyrosine kinase with immunoglobulin (Ig) and epidermal growth factor (EGF) homology domains type 2^+^ (TIE2^+^) M2-like TAMs, and support M1-like macrophages [166]. This key process was modulated by engagement of CD1d, first apoptosis signal receptor (FAS), and CD40 molecules [166] and, of note, iNKT cell transfer into tumor-bearing mice resulted in tumor growth inhibition and decreased M2-like TAMs [166].

### Monocytes/Macrophages

In TIME, the cellular subset recognized as TAMs represents the major component of immune system and plays a crucial role in shaping TIME and in both contrasting and contributing to tumor progression by modulating anti-tumor adaptive immune response, angiogenesis, growth and survival of cancer cells and metastasis formation [167]. Among TAMs two main polarized phenotypes are recognized: the classically activated M1-like and the alternatively activated M2-like that respectively expressed HLA-DR, CD80/86, and CD206, CD163, CD204, stabilin-1 [167, 168]. As commonly accepted, M1-like TAMs exert anti-tumoral activities improving activation of adaptive immune response, while M2-like TAMs support tumor growth by immune suppression, angiogenesis induction and metastasis promotion [167, 168].

Cancer cell can escape the local immune control, giving origin to clones that can recruit circulating monocytes which play a crucial role in metastasis development and reprogram TAMs toward a M2-like phenotype [167, 169].

As for many cancer types, inflammation is a driver in carcinogenesis. In PCa, TAMs are considered central modulators of malignant progression, metastasis formation and therapeutic response [170], thus different studies focused on the clinical and pathological significance of TAMs in prostate tissue.

The M2-like TAM phenotype is driven by different stimuli within TME which include PGE2 [171], CCL2 [also known as monocyte chemoattractant protein 1 (MCP1)] produced by both cancer cells and CAFs in PCa [172], CSF-1 as well as GM-CSF and TGFβ produced by cancer and stromal cells which strongly contribute to macrophages polarization and immunosuppressive environment formation [173], CXCL12 and IL-6 (Figure 2P) [174]. This dialogue from cancer to macrophages is also maintained in the opposite direction as TAMs promote cancer progression stimulating migration and invasion by CCL22-CCR4 axis activation (Figure 2Q) [175]. Also, in *PTEN* null mouse model of PCa, high fat diet (HFD) mediates inflammation and induce M2-like phenotype switching with increased number of CD206^+^ TAMs [176]. Moreover, increased TAM-derived IL-6 pushes PCa growth upon STAT3 pathway activation [177]. This effect is reduced by colecoxib treatments only in mice fed HFD which showed reduced tumor growth and IL-6 production [177].

STAT3 is a key factor involved in CCL5 effect on PCa cells. TAMs-derived CCL5 activates STAT3 signaling in cancer cells and increases cell migration, EMT and cell invasion as well as supports cancer stem cell self-renewal (Figure 2R) [178]. Silencing of CCL5 in TAMs suppressed PCa xenograft growth and bone metastasis formation as tumorigenicity of PCa stem cell *in vivo* [178]. Finally, in human, CCL5 expression correlates with Gleason score, poor prognosis, and metastasis formation [178]. Another mechanism that mediates cancer cell-macrophages crosstalk is driven by the recepteur d’origine nantais (RON) receptor [macrophage stimulating 1 receptor (MST1R)] which is a member of mesenchymal-epithelial transition factor (MET) family of receptor tyrosine kinases [179]. RON is overexpressed on PCa epithelial cells, and its expression correlates with poor prognosis and therapy resistance [179]. RON expressed by cancer epithelial cells mediates tumor growth and metastasis development by modulating macrophage phenotype toward the M2-like. Indeed, the loss of RON, selectively on prostate epithelial cells, induces transcriptional reprogramming on macrophages to support M1-like markers expression [179].

Analysis of 131 biopsies of Japanese PCa patients reveals a positive association between abundance of CD68^+^ macrophages infiltrating the tumor mass and both serum level of PSA [180] and Gleason score [180]. Same conclusion derived from a cohort of 85 patients with prostate carcinoma from a Swedish study in which higher Gleason score correlates with increased density of CD68^+^ macrophages which also results as predictor of shorter cancer-specific survival (CSS) [181].

Another association from the Japanese cohort involves TAMs count and the relapse-free survival rate, which is lower in patients with higher TAMs infiltration [180]. In an American study with 81 PCa patients, TAM density within tumor area positively correlates with Gleason score [182] as confirmed in a Turkish study involving 100 patients in which density of CD68^+^ TAMs infiltration even correlates with tumor stages, extracapsular extension and perineural invasion [183]. The positive association between Gleason score and TAMs number is further confirmed by tissue microarray analysis of 322 prostatectomy specimens in an American cohort in which the greater amount of CD68^+^ macrophages is detected in malignant areas in comparison to healthy tissues [184] and in a German cohort of over 400 patients [185]. An interesting mechanism in PCa-macrophages crosstalk involved semaphorin 3A (SEMA3A) which is produced by cancer cells and recruit monocytes to the tumor site where acquire a pro-tumoral CD68^+^ M2-like phenotype [186]. In this study, it has also been demonstrated that the increased expression of SEMA3A and number of CD68^+^ TAMs negatively correlate with disease-free survival times and disease recurrence [186].

Finally, a microarray analysis comprising 9,393 samples from PCa patients demonstrates that the expression of TAMs-related signature is strongly associated with worse metastasis-free survival [187]. Of note, in a Norway cohort of 59 PCa patients, an increased count of CD68^+^ macrophage is observed in metastasis from lymph nodes, rectum, liver, and bladder as compared to primary tumors [188], suggesting a primarily involvement of macrophages not only in PCa progression but in metastasis formation and development.

Clinical and pathological features of PCa patients displayed association not only with cell count but also with specific macrophage subtypes as results from an Italian cohort of 93 patients in which the amount of CD163^+^ TAMs are associated with extracapsular extension [189]. Increased infiltration of CD163^+^ TAMs also correlates with higher Gleason score (ranging from 8 to 10) as observed in two Swedish studies [190, 191] and the risk of death is twofold higher in patients with high infiltration of CD163^+^ TAMs as compared to those with a lower number of infiltrating TAMs [191].

A novel TAM biomarker, chitinase-3-like protein 1 (CHI3L1, also known as YKL-40 enhances inflammation and angiogenesis within TME [167] and it has been detected at higher concentration in sera of 153 patients with metastatic PCa as compared to healthy subjects [167]. Moreover, in the same cohort of cancer patients, elevated plasma levels of YKL-40 at the time of diagnosis are predictor of a shorted OS [167].

The influence of TAMs is also exerted on the therapeutic response of PCa patients as suggested by multiple evidence. Comparing hormone naïve and CRPC patients, the latter showed an increased number of CD68^+^ TAMs expressing cathepsin S which is involved in ECM remodeling and angiogenesis [192]. Analyzing surgery-derived specimens of pre-treated (cyproterone or leuprolide in combination with flutamide) and untreated patients, the first group displayed increased number of CD68^+^ TAMs [193], similarly to the increased amount of CD68^+^ and CD163^+^ TAMs observed in another cohort of pre-treated patients with Bicalutamide-based androgen deprivation therapy (ADT) [194] or with hormone ablation-treated patients (luteinizing hormone/releasing hormone-agonists and/or antiandrogen prior to surgery [195]. Also, the serum level of YKL-40 could also be considered as prognostic factors for CRPC management thus, the increasing of YKL-40 post-treatment is an independent prognostic factor of early death [193] and of shorter OS [196]. Moreover, another study with 362 PCa patients showed that subject with high M2-like TAMs infiltration displayed the worst prognosis and clinical features and the poorer response to the anti-PD-L1 treatment [197]. These data confirmed an active role of TAMs in modulating PCa progression and disease development also in relation to the adopted therapy and pointed TAMs as promising target to prevent disease recurrence and to improve patient outcomes.

## Conclusion

Immunotherapy has revolutionized the therapeutic approach to cancer, placing the TIMEs as a relevant target for single agent and combination therapy able to reawaken the dormant, anergic immune cells infiltrating tumor tissues. Several strategies have been developed, that include immunocytokine therapies, adoptively transferred cell therapies, generation of chimeric antigen receptor-engineered T (CAR-T) cells and most recently CAR-NK cells. Some of these approaches resulted in relevant progress in cancer treatments, particularly in patients with hematological and some solid (melanoma, lung) cancers. Therefore, a relevant window of failure still persists in the field of immunotherapy, due to the tumor intrinsic and tumor extrinsic features of cancers. Tumors can limit the success of immunotherapy, and in particular in PCa, due to the high heterogeneity of the TME and the TIIME. As a relevant example in the field in this complex scenario, the plasticity of the immune cells, defined as their ability to adapt to the surrounding pathophysiological environment, still represent a challenge. This will culminate to the ability of tumor cells and TME to polarize immune cells, independently from their activation and differentiation state. This latter clearly suggests that an even more precise knowledge of the cellular and molecular mechanisms governing the immune cell response to cancers (e.g., immune cells polarization, immune cells/TME crosstalk) still urges, as a clinical unmet need, to better design successful and personalized immunotherapeutic approaches, to be combined with chemo/radio or targeted therapy and overcame tumor immune/escape and therapy resistance in PCa.

## Abbreviations

AR: androgen receptor
CCL2: C-C motif chemokine ligand 2
CCR4: C-C motif chemokine receptor 4
cDCs1: conventional dendritic cells 1
CRPC: castration-resistant prostate cancer
CSF-1: colony stimulating factor 1
CXCL8: C-X-C motif chemokine ligand 8
CXCR4: C-X-C motif chemokine receptor 4
DCs: dendritic cells
ECM: extracellular matrix
EMT: epithelial-to-mesenchymal transition
ERK1/2: extracellular signal-regulated kinase 1 and 2
IFNγ: interferon gamma
IL-6: interleukin-6
IL-6R: interleukin-6 receptor
iNKT: invariant natural killer T
JAK: Janus kinase
M2: type-2 macrophage
mCRPC: metastatic castration-resistant prostate cancer
MDSCs: myeloid-derived suppressor cells
MICA/B: major histocompatibility complex-class I chain related proteins A and B
M-MDSCs: monocytes-myeloid-derived suppressor cells
MMPs: matrix metalloproteases
NK: natural killer
NKG2D: natural killer group 2D
NKT: natural killer T
NLR: neutrophils-to-lymphocytes ratio
OS: overall survival
PCa: prostate cancer
PD-L1: programmed death-ligand 1
PMN: polymorphonuclear
*PPARδ*: peroxisome proliferator activated receptor delta
PSA: prostate-specific antigen
RON: recepteur d’origine Nantais
Siglec: sialic acid binding immunoglobulin like lectin
STAT: signal transducer and activator of transcription
TAMs: tumor-associated macrophages
TANs: tumor-associated neutrophils
TCR: T cell receptor
TGFβ: transforming growth factor-beta
Th2: T helper 2
TIIME: tumor innate immune microenvironment
TIME: tumor immune microenvironment
TLR9: Toll-like receptor 9
TME: tumor microenvironment
TRAMP: transgenic adenocarcinoma of the mouse prostate
Treg: T regulatory
ULBPs: UL16 binding proteins
VEGF: vascular endothelial growth factor
α-GalCer: alpha-galactosylceramide

## Declarations

### Copyright

© The Author(s) 2022.
